# What Do We Know About the Chevrel Technique in Ventral Incisional Hernia Repair?

**DOI:** 10.3389/fsurg.2019.00015

**Published:** 2019-04-17

**Authors:** Ferdinand Köckerling

**Affiliations:** Department of Surgery and Center for Minimally Invasive Surgery, Academic Teaching Hospital of Charité Medical School, Vivantes Hospital, Berlin, Germany

**Keywords:** Chevrel technique, onlay technique, incisional hernia, recurrence, surgical site occurrence

## Abstract

**Introduction:** In publications on ventral incisional hernia repair, the Chevrel technique and the onlay operation are often equated. This present review now aims to present the difference between these surgical techniques and analyze the findings available on the Chevrel technique.

**Materials and Methods:** A systematic search of the available literature was performed in January 2019 using Medline, PubMed, Scopus, Embase, Springer Link, and the Cochrane Library, as well as a search of relevant journals, books, and reference lists. Thirty-four publications were identified as relevant for this review. For assessment of the Chevrel-technique with other surgical procedures there are no randomized controlled trials, prospective or retrospective comparative studies available but only case series. In the majority of case series the follow-up procedure is not reported.

**Results:** In the onlay technique the defect is closed with direct suture or it is omitted altogether. Whereas, in the Chevrel technique this is done with sliding myofascial flaps harvested from the rectus sheaths. In the few case series available this appears to result in a lower recurrence rate for the Chevrel technique compared with the onlay technique. However, the rates of postoperative complications, surgical site occurrences (SSOs), surgical site infections (SSIs), seroma, and skin necrosis are as high as in the onlay technique. The reason for this is that both techniques require subcutaneous undermining with severance of perforator vessels.

**Conclusion:** If mesh placement in onlay position has been chosen for specific reasons, preference can be given to the Chevrel technique over the standard onlay technique, although the study quality is limited.

## Introduction

In publications on ventral incisional hernia repair, the Chevrel technique and the onlay operation are often equated (1, 2). Onlay repair places the mesh on the anterior fascia, which typically involves dissection and primary closure of the fascia or a bridging situation below the mesh (3–5). By contrast, in the Chevrel technique the defect is first closed tension free through overlapping herniorrhaphy and then reinforced with an onlay prosthetic implant (6, 7). Because of defect closure with sliding myofascial flaps harvested from the incised anterior layers of the rectus sheaths, the Chevrel technique differs greatly from direct defect closure or bridging as used in the onlay technique (8, 9). Therefore, these different techniques should be clearly distinguished from each other and analyzed separately (5, 8, 9).

This review now summarizes the publications available to date for the Chevrel technique and analyzes the findings. The historical evolution of this technique and its variations will also be discussed.

## Materials and Methods

**Figure 1:**
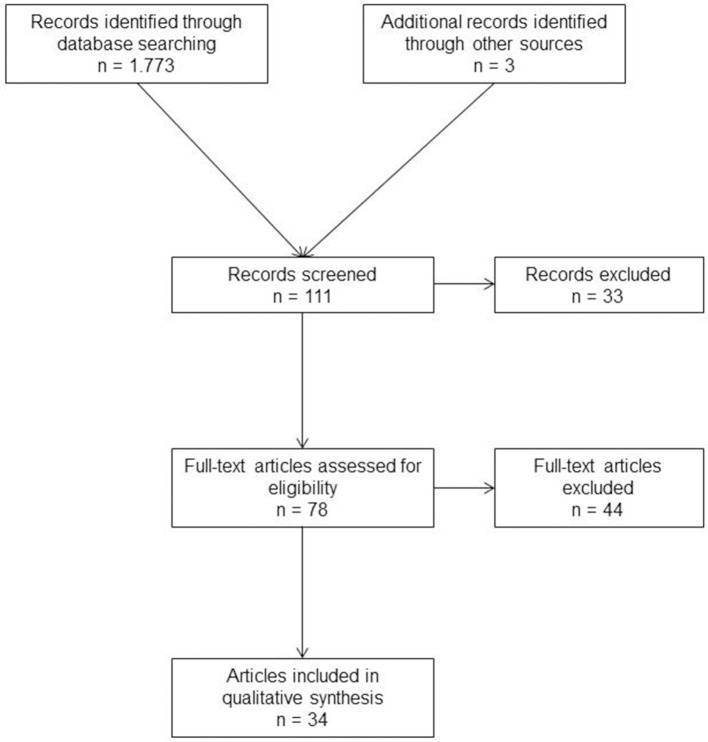
Prisma flow diagram of study inclusion.

A systematic search of the available literature was performed in January 2019 using Medline, PubMed, Scopus, Embase, Springer Link, and the Cochrane Library, as well as a search of relevant journals, books and reference lists. The following search terms were used: “Chevrel technique,” “Onlay technique,” “incisional hernia and onlay technique,” “incisional hernia and Chevrel's technique.” The abstracts of 111 publications were screened (Figure 1). Only case series and no comparative randomized controlled trial, prospective, and retrospective studies were available. The published case series contained mostly insufficient information concerning the follow-up procedure. For the present analysis, 34 publications were identified as relevant for the key question (Figure 1). A systematic presentation and synthesis of the characteristics and findings of the included studies have been made in accordance with the Prisma guidelines (10) (Table 1).

**Table 1:** Incisional hernia repair in Chevrel technique.

**References**	**Hernia type**	**Study type**	***n***	**Mesh**	**Postoperative complications**	**Recurrence rate**	**Follow-up procedure**	**Follow-up**
Whiteley et al. (11)	Incisional	Case series	10	Polypropylene	0%	0%	Not reported	Median 17 months
Khaira et al. (12)	Incisional	Case series	35	Polypropylene, fascia	Seroma 17%, hematoma 3%, superficial wound infection 3%	0%	Clinic visits and telephone surveys	Median 20.3 months (range 6.0–54.1 months)
Chevrel (13)	Incisional	Case series	143	Prolene, Mersilene, Dexon	Total 10.5%, seroma 6.3%, hematoma 1.4%, superficial infection 2.8%	4.9%	Not reported	1–20 years
Schug-Pass et al. (14)	Incisional	Case series	39	TiMesh, Atrium, Parietene	–	5.1%	Clinical and ultrasound examination	Mean 25.8 months (range 6.5–53.3 months)
Licheri et al. (15)	Incisional	Case series	64 (45 elective, 19 emergency)	Polypropylene, polyester, polypropylene + polyglactin	26.5%	3.0%	Not reported	Mean 54 months (range 4–120 months)
Kaafarani et al. (16)	Incisional	Case series	86 (10 converted from laparoscopic)	Polypropylene	Seroma 23.3%	–	No follow-up longer than 8 weeks	8 weeks
Joshi et al. (17)	Incisional	Case series	30	Polypropylene	13.3%	0%	Not reported	Minimum follow-up 12 months
Mladenovikj et al. (18)	Incisional	Case series	319	Polypropylene	SSI 17%, partial skin necrosis 4%	2.1%	Not reported	6–48 months
Mommers et al. (19)	Incisional	Case series	155	Prolene	SSO 19.4%, seroma 10.3%	1.8%	Not reported	Median follow-up 52 months (range 12–128 months)
Hodgman and Watson (20)	Incisional	Case series	123	Polypropylene 81%, biologic mesh 19%	Seroma 21%, Skin breakdown 30%	5.1%	Not reported	Median 17.8 months (range 0.3–134.6 months)

## Results

### Evolution of the Chevrel Technique

In 1929, Gibson first described a tissue hernia repair using the anterior rectus sheath by relaxing incisions for tension free defect closure (21). According to Gibson the main principle of the operation is to close the gap chiefly by approximating the refreshed edges of the rectus sheath, tension being relieved by relaxing incisions parallel to the line of suture on either side (21).

Rothschild described in 1933 a procedure which “was done through a misunderstanding of the Gibson technique” (22). After cleaning of the anterior rectus sheath of all adipose tissue, transverse incisions above and below are made in the sheath and these transverse incisions are connected by longitudinal incisions. These lateral flaps are raised and hinged along their medial border (22). They are then sutured by a continuous stitch (22).

Dixon reported in 1929 his addition of a shoelace suture technique (23). The shoelace operation reconstructs a strong new linea alba, straightens the rectus muscles to lie side by side at the midline, reconstructs the anterior rectus sheath by a long suture, and fixes them to the new linea alba (23). For the shoelace suture, a 6 m length of No. 0 or 1 monofilament polyamide is used by Abrahamson, doubled to form a loop 3 m long (24).

The procedure of Welti and Eudel was used in France for midline repair and consists of making two lateral incisions parallel to the midline through the anterior rectus sheath (25). The two resulting medial aponeurotic flaps are then rotated medially and sutured together over the midline defect (25).

Rehn described for the first time in 1957 his cutisplasty (26) where for incisional hernias the defect is closed at both sides with segments harvested from the anterior layer of the rectus sheath. The two medial segments of the rectus sheath are sutured together at the midline to create a new linea alba. The defect in the anterior layer of the rectus sheath with exposed rectus muscles is replaced with a cutisplasty harvested from the thigh (26). Hence, the cutisplasty reinforces the abdominal wall reconstruction by relieving tension of the median gathering sutures, resulting in anatomic reconstruction (26).

In 1979, Chevrel in France, Browse in the UK and Deitel in Canada further modified the anterior rectus sheath repair by adding an “overcoat” of synthetic mesh, thus recreating the anterior rectus sheath and reinforcing the midline repair (6, 13, 27, 28).

Browse described his technique for the repair of large midline abdominal incisional hernias using reflected flaps of anterior rectus sheath reinforced with Marlex mesh (27). Deitel and Vasic (28) used the technique described by Gibson (21) with longitudinal incisions through the anterior rectus sheath on each side for relieve of tension. A Marlex mesh is trimmed to a properly fitting ellipse and the margins of the mesh are sutured securely to the lateral margins of the relaxing incisions (28).

Chevrel reported in 1979 about 12 cases with good functional results having used a combination of an “overcoat” plasty of the anterior layer of the rectus sheaths, reinforced by a pre-muscular Mersilene patch (6).

In 1997, Chevrel and Rath (7) published details of a series of operations for incisional hernia treated either by primary suture or by a plasty reinforced with a Dacron (Mersilene®) or Polypropylene (Prolene®) mesh placed anterior to the rectus sheath, and fixed by a new method involving a spray of fibrin glue. The use of a prosthesis fixed with fibrin glue reduces the definitive recurrence rate to 0.97% against 9.02% for techniques without a prosthesis in an overall series of 389 operations (7).

### Surgical Technique

**Figure 2:**
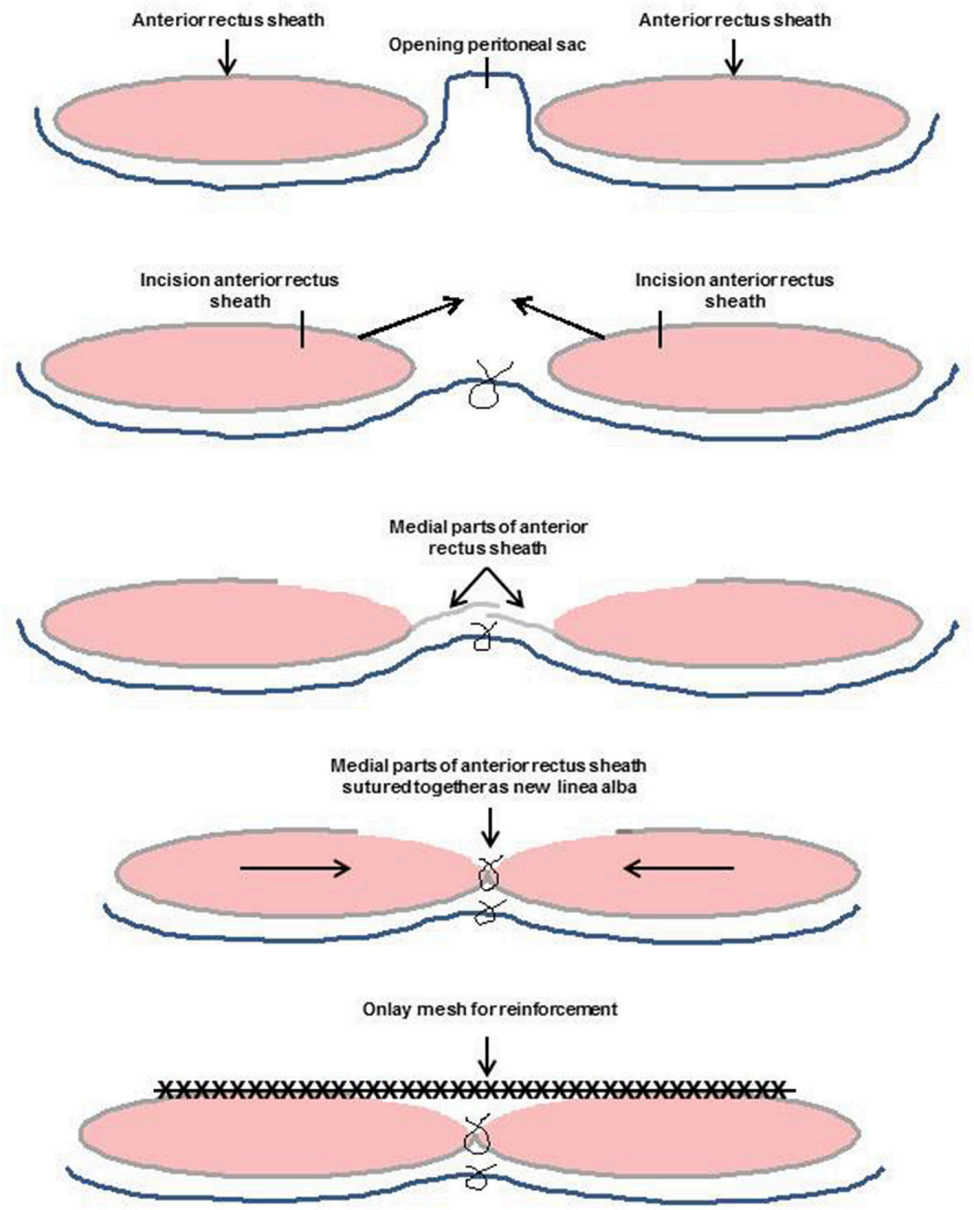
Incisional hernia repair in Chevrel technique.

In 2001, Chevrel once again demonstrated his technique in detail (13). Following complete excision of the scar, the hernia sac is dissected and resected (Figure 2). The anterior layer of the rectus sheath is exposed at both sides through resection of the skin and subcutaneous tissue. The anterior rectus sheaths are vertically incised 2 cm from their medial borders, creating two medial strips that are freed on their undersurfaces from the recti, mainly at the fibrous intersections that cross the recti at three or four levels (13). Several perforating vessels will be identified, and careful hemostasis must be observed (13). The peritoneum is closed with a continuous absorbable suture (13). The medial edges of the defect are now approximated with 2-0 non-absorbable sutures (13). The new linea alba is then sutured with two rows of interrupted non-absorbable u-sutures (13). This plasty is then reinforced by a prosthesis that is anchored 3–4 cm lateral to the medial border of the remaining rectus sheath (13).

## Results Obtained With the Chevrel Technique

Whiteley et al. (11) in 1998 published a series of 10 cases with large incisional hernias repaired in the Chevrel technique using polypropylene meshes. No wound complication and no recurrence (median follow-up of 17 months) was seen.

Khaira et al. (12) in 2001 performed 35 incisional hernia repairs in the Chevrel technique using polypropylene mesh. Postoperative complications included seroma formation in six patients, deep vein thrombosis in one, a non-fatal pulmonary embolism in another. One patient developed a wound hematoma and one had a superficial wound infection (12). Follow-up was a median of 20.3 months (range 6.0–54.1 months). Two of these (6%) patients reported a persistent lump and one (3%) reported persistent pain, but none was found to have a recurrence (12).

In 2001, Chevrel (13) himself published a case series on 143 incisional hernia operations in his technique. Ninety three percent of his patients were followed up for one to 20 years. The overall morbidity rate was 10.5%. There were two hematomas (1.4%), nine seromas (6.3%) and four superficial infections (2.8%). Seven recurrences had been noted (4.9%).

Schug-Pass reported in 2006 (14) on 39 incisional hernia repairs in the Chevrel technique. In a median follow-up of 25.8 months (range 6.0–54.1 months) the recurrence rate was 5.1%.

Licheri et al. (15) in 2008 reported on a case series of 64 midline incisional hernia repairs in Chevrel technique. Nineteen were operated in emergency and 45 electively. Prosthetic material was polypropylene (53%), polyester (42%), and polypropylene and polyglactin 910 (5%). The mortality rate was 1.8%. Postoperative complications were exclusively parietal in 17 patients (25.5%), i.e., seroma, skin necrosis and superficial wound infection. No deep infection or intra-abdominal complications were observed (15). In a mean follow-up of 54 months (range 4–120 months) two recurrences (3%) were detected (15).

In a prospective randomized trial at four Veterans Affairs hospitals with 86 incisional hernia repairs in Chevrel technique and 59 laparoscopic IPOM technique the seroma rate for the open approach was 23.3% (16).

Joshi et al. (17) in 2015 published a series of 30 patients with midline incisional hernia treated with the Chevrel technique using a polypropylene mesh for reconstruction of the anterior rectus sheath. Immediate postoperative complications were seen in 13.33% of patients. The recurrence rate was zero at a minimum follow-up of 12 months (17).

Mladenovikj et al. (18) in 2016 reported their experiences with 319 patients with midline giant incisional hernias electively operated in the standard Chevrel technique with the creation of a new linea alba. The defect on the anterior rectus sheath was covered with polypropylene mesh. Surgical site infections were rated in 56 (17%) patients. Seroma was a leading complication in this series. Partial necrosis of the skin was seen in 12 patients (4%). Hernia recurrence was observed in 7 patients (2.1%) during the follow-up period (6–48 months) in the controlled 284 patients.

Mommers et al. (19) published in 2017 a series of 154 patients operated in the Chevrel technique from 1979 (6). This technique does not require such large subcutaneous dissection, since the mesh is sutured to the remnant of the anterior rectus sheath with onlay one-and-a-half centimeters overlap (6, 19). Within 30 days postoperative 36 patients (23.2%) had 39 postoperative complications, of which 30 were mild, and nine severe (19). Thirty-one surgical site occurrences (SSOs) were observed in thirty patients (19.4%), of which the majority were seroma (16 patients; 10.3%). The recurrence rate was 1.8% after a median follow-up of 52 months (12–128 months) (19).

Hodgman in 2017 published a series of 123 patients with incisional hernia repair in Chevrel technique (20). Twelve patients had a lateral component release in addition to release of the anterior rectus sheath (20). In 81% of the patients a synthetic and in 19% a biologic mesh was used (20). Seroma formation in 21% and skin breakdown in 30% were the most common complications (20). The recurrence rate for patients with a follow-up of more than 36 months was 7% (20).

## Discussion

The Chevrel technique used to repair incisional hernias differs in terms of operative details from the onlay technique and should therefore be analyzed separately. Whereas, in the onlay technique the defect is closed with direct suture or left open as a bridging (5), in the Chevrel technique the defect is closed with sliding myofascial flaps obtained from incision of the anterior layers of rectus sheaths (6, 7). A common feature of both techniques is placement of the mesh in the onlay position on the fascia (5–7).

Compared with the onlay technique, the Chevrel operation appears to have a lower recurrence rate. In a maximum follow-up of 52 months the recurrence rate was 0–7% with a mean value of 2.7%.

The onlay technique has a range of 0–32% and a mean value of 9.9% (5). That difference can most likely be explained by the more tension-free defect closure in Chevrel technique. Hence, in that respect the Chevrel technique appears to be superior to the onlay technique. Myofascial defect closure reduces the recurrence rate compared with direct defect closure or a bridging situation.

Surgical site infections and seromas are the Achilles heel of all types of abdominal wall hernia reconstruction (29). As in the onlay technique, high rates of surgical site infections and seromas are also seen in the Chevrel technique.

The literature reports seroma rates of up to 21% and surgical site infection and skin necrosis rates of up to 30% (20). That somewhat corresponds to the wound complication rates reported for the onlay technique (5). Hence, when applying that outcome criterion, no relevant difference is seen between the onlay and Chevrel techniques.

In the case of onlay or Chevrel mesh repair of an incisional hernia, significant subcutaneous undermining is necessary to place the mesh and achieve adequate overlap (30). Skin undermining of more than 2 cm has been shown to increase the risk of SSOs (30). Maximal perforator preservation has been shown to reduce the rate of wound healing complications (30).

Other measures that can help to avoid seromas after Chevrel repair include drains (31, 32), wearing an abdominal binder (33) and fixation of the skin-subcutaneous flaps to the mesh with fibrin glue at the end of the operation (34). But sufficient evidence is not available to date for any of these measures. Limitation of this review is the non-availability of comparative data. Additionally, the existing case series mostly contain no sufficient information concerning the follow-up of the patients.

In summary, it can be stated that compared with the onlay technique, the Chevrel technique for ventral incisional hernia repair appears to have a lower recurrence rate. That can be explained by the more tension-free defect closure with myofascial sliding flaps harvested from the rectus sheaths. However, as in the onlay technique, for mesh reinforcement subcutaneous undermining with severance of perforator vessels is needed, thus resulting in reduced perfusion of the skin flaps. That is thought to be the reason for the higher incidence of SSOs (surgical site infections, seroma, skin necrosis) identified for mesh placement in the onlay position. Therefore, preference should be given to techniques without the need for creation of skin flaps (sublay, transversus abdominis release) over the onlay and Chevrel techniques. Nonetheless, these techniques too may have advantages under certain conditions. In this case, all measures should be taken to reduce the risk of postoperative complications.

## Author Contributions

The author confirms being the sole contributor of this work and has approved it for publication.

### Conflict of Interest Statement

The author declares that the research was conducted in the absence of any commercial or financial relationships that could be construed as a potential conflict of interest.
